# Advanced Genetic Studies on Powdery Mildew Resistance in TGR-1551

**DOI:** 10.3390/ijms232012553

**Published:** 2022-10-19

**Authors:** María López-Martín, Ana Pérez-de-Castro, Belén Picó, María Luisa Gómez-Guillamón

**Affiliations:** 1 COMAV, Instituto de Conservación y Mejora de la Agrodiversidad, Universitat Politècnica de València, Cno. de Vera, s/n, 46022 València, Spain; 2 IHSM La Mayora, CSIC-UMA, Avda. Dr. Wienberg s/n, 29750 Málaga, Spain

**Keywords:** powdery mildew, molecular markers, dominant–recessive epistasis, marker-assisted selection, TIR-NBS-LRR

## Abstract

Cucurbits powdery mildew (CPM) is one of the main limiting factors of melon cultivation worldwide. Resistance to races 1, 2, and 5 has been reported in the African accession TGR-1551, whose resistance is controlled by a dominant–recessive epistasis. The dominant and recessive quantitative trail loci (QTL) have previously been located in chromosomes 5 and 12, respectively. We used several densely genotyped BC_3_ families derived from the cross between TGR-1551 and the susceptible cultivar ‘Bola de Oro’ to finely map these resistance regions. The further phenotyping and genotyping of the selected BC_5_, BC_5_S_1_, BC_5_S_2_, BC_4_S_1_, BC_4_xPS, and (BC_4_xPS) S_1_ offspring allowed for the narrowing of the candidate intervals to a 250 and 381 kb region in chromosomes 5 and 12, respectively. Moreover, the temperature effect over the resistance provided by the dominant gene has been confirmed. High resolution melting markers (HRM) were tightly linked to both resistance regions and will be useful in marker-assisted selection programs. Candidate R genes with variants between parents that caused a potential modifier impact on the protein function were identified within both intervals. These candidate genes provide targets for future functional analyses to better understand the resistance to powdery mildew in melons.

## 1. Introduction

Melons (*Cucumis melo* L.) constitute an economically important vegetable crop. In 2019, their world production reached 27,5 million tons [1] (http://faostat3.fao.org accessed on 1 June 2022). Among the most important diseases affecting this crop, cucurbit powdery mildew (CPM), a fungal disease occurring in both field and greenhouse conditions worldwide, reduces its productivity. The occurrence of CPM early in the growing season can lead to a massive reduction in photosynthetic capacity, with negative impacts on plant growth and development, as well as fruit quality [2,3,4]. Two ectoparasites, *Golovinomyces orontii* (*Go*) and *Podosphaera xanthii* (*Px*), are economically important and distributed worldwide [5], as they are both the two most commonly reported causal agents of CPM [6,7].

Spatiotemporal changes in the geographic distribution of both Go and *Px* have been observed during the last three decades [3,4,6]. Significant variations in virulence, expressed by numerous pathotypes and/or races and their spatiotemporal fluctuations, have been described for both species [2,3,4,8,9,10,11]. De Miccolis Angelini et al. [12] emphasized that *Px* has great potential to evolve new, better-adapted genotypes that can overcome the currently used resistance genes and the efficacy of modern fungicides. Variability in *Px* virulence, recorded as different physiological races, was first observed in the U.S. in 1938, when race 2 appeared shortly after the release of race 1-resistant ‘PMR 45′ produced in Imperial Valley, California [13]. Since then, many physiological races have been identified according to the reactions after infection of a set of differential melon lines, and several cultivars with resistance to some of those races have been described.

To date, several genes and QTL associated with resistance to CPM have been mapped in different melon populations derived from several genetic sources, including *Pm-x* [14], *Pm-x1,5* and *Pm-x3* [15], *Pm2F* [16], QTL (AR5) [17], and *Pm-Edisto47-2* [18] on chromosome 2; *qPx1-4* [19] on chromosome 4; *Pm-w* [20], *Pm-R* [21], *Pm-AN* [22], *qPx1-5* [19], and QTL *PmV-1-Pi124112* [23] on chromosome 5; *CmPMrs* [24] and *qPx1-10* [19] on chromosome 10; *Pm-y* [14], QTL *PmXII-1-Pi124112* [23], QTL (AR5) [17], *Pm-Edisto47-1* [18], *CmPMR1* [24], *qPx1-12* [19], *qCmPMR-12* [25], and *BPm12.1* [26] on chromosome 12; and *Pm-1* [27] on LGIX. Most of these resistances are race-specific and controlled by one or two dominant genes. Epistatic effects have been described between *CmPMrs* and *CmPMR1* QTLs [24], as well as between *qPx1-5* and *qPx1-12* [19]. In addition, several studies reported that Mildew Locus O (MLO) genes act as susceptibility factors in CPM disease and their inactivation of specific MLO genes (knock-out or knock-down) leads to a mediating form of *mlo* resistance [28,29]. Natarajan et al. [30] carried out a comparative analysis of 14 MLO genes and investigated their SNIPs and InDels variations, finding associations among them.

Among the resistant cultivars described, the Zimbabwean melon TGR-1551 carries resistance to races 1, 2, and 5 of *Px*. The inheritance of such resistance was studied in a cross of TGR-1551 with the Spanish susceptible cultivar ‘Bola de Oro’ and the results indicated that it was governed by two independent genes, one dominant and one recessive, whose genetic control corresponded to dominant–recessive epistasis [31]. A QTL analysis carried out on the F_2_ generation derived from the initial cross allowed for the identification of one major QTL (*Pm-R*) on chromosome 5 for resistance to the three races [32]. A RIL population (F_7_:F_8_) obtained from that cross was also evaluated for *Px* resistance to races 1, 2, and 5, and QTL analyses carried out by Beraldo-Hoischen et al. [33] confirmed the existence of the *Pm-R* QTL in chromosome 5, associated with the dominant gene. An additional QTL, possibly associated with the recessive gene, was also detected for *Px* resistance. This QTL is located on chromosome 12 and several microsatellite markers (TJ29, CMBR111, and CMBR150) were described as being associated with resistance [33]. Two QTL associated with resistance to *Px* had been previously identified on chromosome 12. One of them is the QTL *PmXII.1* [23], associated with the *Pm-y* gene and controlling resistance to races 1, 2, and 5 derived from PI 124112. The direct association of *PmXII.1* with the minor QTL reported in TGR-1551 was not possible due to the lack of common molecular markers between both genetic maps [33]. The other QTL was identified by Fukino et al. [17] and associated with one of the two dominant genes for resistance to race 1 described previously in PMR 5 [34], as well as with *PmXII.1*. This QTL from Fukino et al. [17] was strongly linked to the microsatellite markers associated with the QTL derived from TGR-1551 (TJ29, CMBR111, and CMBR150) [33]. This candidate interval proposed by Fukino et al. [17] has recently been confirmed by Hong et al. [35] and the resistance of MR-1 to race 1 has recently been mapped to the same region [19,24]. More recently, Howlader et al. [36] described three SNP markers associated with specific resistance to race 5 of *Px* in chromosome 12, in close proximity to the microsatellite markers previously described by Fukino et al. [17] and Beraldo-Hoischen et al. [33]. The recessive gene present in TGR-1551 is unique, since no recessive genes conferring resistance to more than one *Px* race has been previously reported in melons. This gene could confer a durable resistance to powdery mildew, similar to the resistance conferred by *mlo* in barley [37]. CPM resistance is an important objective of melon-breeding programs, which implies the identification of molecular markers and possible candidate genes associated with the trait [38].

The objective of this work was to finely map the recessive locus derived from TGR-1551 conferring resistance to *Px* by analyzing the advanced backcross populations segregating only at the target genomic region associated with the recessive gene previously described. In addition, a fine mapping of the genomic area where the dominant resistant gene is located in chromosome 5 has been carried out. The generations used in this study were constructed from an original BC_3_F_1_ (200 families) obtained after three successive backcrosses to the susceptible cultivar ‘Bola de Oro’ (BO).

## 2. Results

### 2.1. Selection of BC_3_, BC_3_S_1_, and BC_4_ Generations

An existing panel of 124 SNP markers evenly distributed throughout the genome (Background markers 1) (Supplementary Table S1) [39,40,41,42,43] were used to genotype 200 plants of the BC_3_ (TGR-1551 x BO derived) population in the Agena Bioscience Platform. The resistance to CPM derived from TGR-1551 had previously been described as being controlled by a dominant–recessive epistasis, [31] where a QTL related to the dominant gene was located in chromosome 5 [21,32,33] and another QTL linked to the recessive gene mapped in chromosome 12 [33]. A total number of 20 BC_3_ plants carrying different regions introgressed in these chromosomes were selected (Table 1). For the rest of the genome, a high percentage of the BO genetic background was prioritized. These selected plants were also genotyped in the Agena Bioscience platform with the markers set as CYSDV1, which contained 16 additional SNP markers located in chromosome 5 (markers set CYSDV1) [44] and with the markers sets WMV1 and WMV2 [43], which had 13 and 3 SNP markers in this region, respectively (Supplementary Table S2) (Table 1). The selfing progenies and BC_4_ offspring (10 to 20 plants) of these 20 plants were genotyped with the set of markers CYSDV1 and CYSDV2 (Supplementary Table S2) [44]. Moreover, those plants were genotyped with the previously developed HRM markers *cysdv63* and *cysdv65* [44]. These generations were used to finely map the regions associated with resistance.

### 2.2. Narrowing of the Candidate Region on Chromosome 5

The 20 BC_3_S_1_ families were phenotyped for the resistance against races 1, 2, and 5 of *Px* (Table 1). The response of the offspring of those plants not carrying a TGR-1551 introgression in chromosome 12 depended on the genotype in chromosome 5. Segregation for the resistance was observed for those families with parents heterozygous for the region between markers *cysdv63* (chr5: 25,782,654 bp) and *CMPSNP464* (chr5: 26,405,006 bp) (BC_3_ plants 19, 64, 159, and 166) (Table 1). Contrarily, all the descendants from parents without a TGR-1551 introgression for this region (BC_3_ plants 28 and 37) were susceptible. Otherwise, BC_3_ plant 198, whose BC_3_S_1_ offspring was susceptible to *Px*, was homozygous for the BO allele at the *locus cysdv63* and heterozygous at the position of the marker *CMPSNP464* (Table 1), setting in this marker the lower limit of the candidate resistance region. Moreover, BC_3_-198 also carried a TGR-1551 introgression at chromosome 12 that seemed to be unrelated to the recessive resistance (Table 1). On the other hand, the family BC_3_S_1_-146 was also susceptible but it segregated at the position of the marker *cysdv63,* whereas it was homozygous for the BO allele at the *locus cysdv65* (chr5: 26,408,895 bp), narrowing the candidate interval to a region of ~622 kb between markers *cysd63* and *CMPSNP464*. This same region had been described as related to CYSDV resistance derived from ‘TGR-1551’ [44].

**Figure 1:**
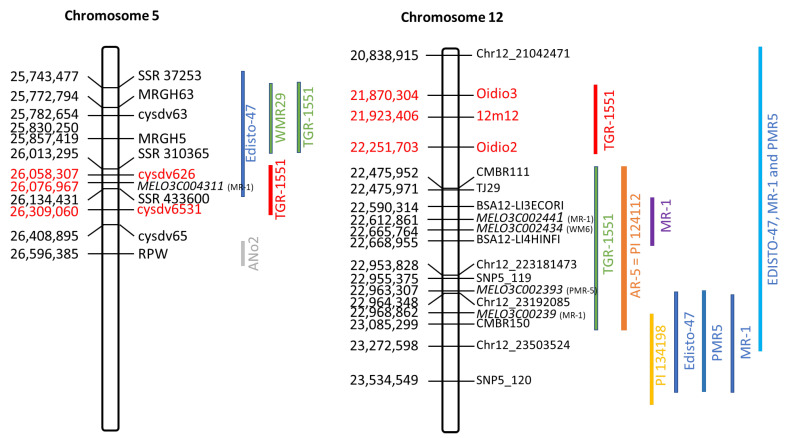
Genomic regions associated with the resistance to powdery mildew (*Podosphaera xanthii*) derived from different sources. The results obtained in this work are marked in red and the previous results derived from TGR-1551 are represented in green. The proposed candidate genes for other resistance sources are also indicated. The presented physical positions are refer to the genome version v.4.0 of *C. melo* (available at https://melonomics.net/ accessed on 1 June 2022).

To narrow the candidate interval in chromosome 5, the heterozygous plants for this region—BC_4_-159 plants 4 and 11—were selected. The plant BC_4_(159)4 was crossed with ‘Piel de Sapo’ (PS) to obtain the BC_4_(159)4xPS offspring. It was also backcrossed to BO to obtain the BC_5_(159)4 progeny. In the case of the plant BC_4_(159)11, only the BC_5_ offspring backcrossed to BO were obtained. These families (approximately six plants each) were genotyped with the HRM markers *cysdv63* and *cysdv65* and those plants that were heterozygous for both markers were selected and genotyped with background markers 2 set in the Agena Bioscience platform. The plants with the higher genetic background for the BO or PS genome were selected and self-pollinated. Families BC_4_(159)4xPS-7S_1_, BC_4_(159)4xPS-18S_1_, BC_5_(159)4-14S_1_, and BC_5_(159)11S_1_ were phenotyped for the resistance to races 1, 2, and 5 of CPM (20 plants per family). They were also genotyped with the HRM markers *cysdv63* and *cysdv65*. The phenotyping/genotyping results of these offspring were compatible with the interval for the dominant gene obtained in the BC_3_ and BC_3_S_1_ analyses. Moreover, three plants were found to be recombinant in the candidate region: (BC_4_(159)4xPS-7S_1_ plants 2 and 13 and BC_5_(159)4-14S_1_ plant 5). These plants were also genotyped with the HRM markers *cysdv626* (chr5: 26,058,307 bp) and *cysdv6531* (chr5: 26,309,060 bp) to narrow the interval (Table 2). These three plants were heterozygous at the position of the marker *cysdv626* and homozygous for the BO allele for the marker *cysdv65*. For the marker *cysdv6531*, BC_4_(159)4xPS-7S_1_ plant 2 was heterozygous, while plants BC_4_(159)4xPS-7S_1_-13 and BC_5_(159)4-14S_1_-5 were homozygous for the BO allele. While the BC_4_(159)4xPS-7S_1_ plants 2 and 13 were resistant to CPM, BC_5_(159)4-14S_1_ plant 5 was susceptible. These phenotyping/genotyping results enabled us to narrow the candidate interval to a region of 250,753 bp between markers *cysdv626* and *cysdv6531* (Figure 1). There are 34 predicted genes in the candidate region (Supplementary Table S3). GBS data were used to perform an SNP-calling analysis and SNPeff was used to predict the effects of genetic variants found between TGR-1551 and BO in this candidate region. SNPs were only found in two candidate genes, *MELO3C004297* and *MELO3C004311*, that coded a branched-chain-amino-acid aminotransferase-like protein and a TMV resistant protein N-like, respectively. The detected SNPs had a modifier-predicted impact on the coded protein functions (Supplementary Table S4). One of these candidate genes, *MELO3C004311*, has also been proposed as the gene conferring resistance in the accession MR-1 [19].

Several BC_5_S_1_ plants, which were homozygous for the TGR-1551 alleles at the candidate region of chromosome 5, were self-pollinated to obtain their BC_5_S_2_ offspring. These BC_5_S_2_ families were phenotyped for resistance against *Px* races 1, 2, and 5 and all of them resulted in being resistant, validating the previously obtained results.

### 2.3. Narrowing of the Candidae Region on Chromosome 12

Eight BC_3_ plants that only had a TGR-1551 introgression in chromosome 12 (BC_3_ plants 34, 78, 88, 96, and 139), or with an introgression not related to CPM resistance in chromosome 5 (BC_3_ plants 24, 89, and 148), were analyzed. The selfing progenies of two of these plants heterozygous for markers *CMPSNP285* (chr12: 20,421,309 bp) and *CMPSNP361* (chr12: 23,000,406 bp) (BC_3_ plants 34 and 139) segregated for the resistance in question, while the descendants of plant BC_3_-88 were susceptible. Four recombinant plants were found according to these two markers (BC_3_ plants 78, 89, 96, and 148) (Table 1). On the one hand, BC_3_-78 was heterozygous for the marker *CMPSNP285* and homozygous for the BO allele for the marker *CMPSNP361* and its offspring segregated for resistance. On the other hand, the BC_3_ plants 89, 96, and 148 were homozygous for the BO allele for the marker *CMPSNP285* and heterozygous for the marker *CMPSNP361* with susceptible, segregant, and susceptible progenies, respectively. The high level of recombination in this region demonstrated that the BC_3_S_1_-88 offspring were susceptible to CPM even though the BC_3_-88 plant was heterozygous for both markers in chromosome 12. These results allowed us to initially narrow the candidate interval to a 2.6 Mb region between markers *CMPSNP285* and *CMPSNP361*.

Remarkably, those BC_3_S_1_ families whose parents had the TGR-1551 introgressions at both candidate regions of chromosomes 5 and 12 (BC_3_ plants 95, 105 and 141) had a higher ratio of resistant plants, which agrees with a dominant–recessive epistatic model.

Based on GBS data previously obtained by the research group, 10 high-resolution melting markers (HRM) were designed to narrow the candidate region in chromosome 12 (Supplementary Table S5). These HRM markers were evenly distributed throughout the interval between markers *CMPSNP285* and *CMPSNP361*. Six BC_3_ plants and their BC_3_S_1_ offspring, which had previously been phenotyped for resistance to CPM (BC_3_S_1_ families 34, 88, 89, 96, 139, and 148), were genotyped with these 10 HRM markers (Table 3). These results showed that a recombination event had taken place within markers *CMPSNP285* and *CMPSNP361* in the BC_3_-88 plant. Only those BC_3_S_1_ families whose parents were heterozygous for both markers *Oidio2* (chr12: 22,251,703 bp) and *Oidio1* (chr12: 22,309,972 bp) (BC_3_S_1_ families 34, 96, and 139) segregated for the resistance to CPM. The plant BC_3_-139 was homozygous for the BO allele at the position of the marker *Oidio3* (chr12: 21,870,304 bp) and its selfing progeny was segregant for resistance, setting the upper limit of the candidate region at the position of the *locus Oidio3*. BC_3_ plants 88 and 148 were homozygous for the BO allele for the markers *Oidio3*, *Oidio2,* and *Oidio1* and heterozygous for the marker *OidioC* (chr12: 22,875,711 bp). Their offspring were susceptible to CPM, narrowing the candidate interval to a ~1 Mb region between markers *Oidio3* and *OidioC*.

The genotyping of the BC3S_1_ families that segregated for CPM resistance showed significative phenotypic differences between those plants that were homozygous for the TGR-1551 allele for the markers *Oidio2* and *Oidio1* and those that were heterozygous or homozygous for the BO allele at these positions.

Afterwards, BC_4_-95 plant 10 was selected, as it was heterozygous at the candidate region of chromosome 5 and at the position of the marker *SNP361*. It was self-pollinated and crossed with the PS cultivar BGCM-126 to obtain the BC_4_(95)10S_1_ and BC_4_(95)10xPS offspring, respectively. Six plants of the BC_4_(95)10xPS offspring were genotyped with a new set of 160 SNP markers evenly distributed throughout the genome (Background marker 2) (Supplementary Table S1). Moreover, these plants were also genotyped with the HRM markers *cysdv63* and *cysdv65* (Table 3). Those plants homozygous for the BO allele at the candidate region of chromosome 5 and heterozygous for the marker *CMPSNP361* at chromosome 12 were selected (BC_4_(95)-10xPS plants 3 and 5). These two plants were also genotyped with the HRM markers *Oidio3*, *Oidio2*, and *Oidio1* (Table 4). Both were homozygous for the BO allele at the position of the markers *Oidio3* and S12_22130778 (background marker 2, chr12: 21,923,406 bp) and heterozygous for markers *Oidio2* and *Oidio1.* Their selfing progenies were evaluated for their resistance to races 1, 2, and 5 of CPM. All the BC_4_(95)-10xPS-S_1_ plants were susceptible, independently of their genotype for the *Oidio2* marker. This allowed us to narrow the candidate interval to a 381,399 bp region between markers *Oidio3* and *Oidio2* (Figure 1). This region had 50 annotated genes, some of which could be good resistance candidates (Supplementary Table S5).

SNPeff was used to annotate and predict the effects of the SNPs found in two GBS assays between TGR-1551 and BO within this region. SNPs were detected in the sequence of 19 candidate genes in this region. Most of those SNPs had a predicted modifier impact (Supplementary Table S4). Ten of the affected genes coded a serine/threonine-protein kinase. SNPs were also detected in genes coding (3S,6E)-nerolidol synthase 1-like (*MELO3C002520*), a eukaryotic translation initiation factor-like protein (*MELO3C002515*), a BTB/POZ domain-containing protein At3g22104 (*MELO3C002514*), a protein detoxification (*MELO3C002526*), purine permease 3-like (*MELO3C002545*), an unknown protein (*MELO3C035727*), and three receptor-like protein kinases (*MELO3C002504, MELO3C002541*, and *MELO3C002538*) (Table 3).

### 2.4. Changes in Resistance Levels Due to Low Temperatures

**Figure 2:**
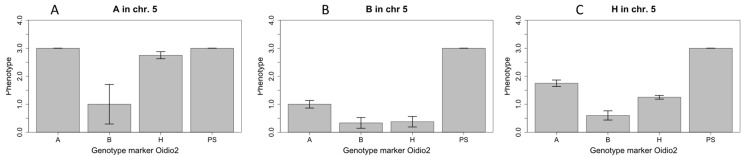
Interaction between QTL on chromosome 5 (markers cysdv63 and cysdv65) and QTL on chromosome 12 (marker Oidio2) for symptom score at 12 days post-inoculation (bars indicate standard error). Genotype for marker Oidio2: BO: homozygous for ‘Bola de Oro’ allele; H: heterozygous: TGR: homozygous for TGR-1551 allele. Genotype for marker cysdv63: A: homozygous for ‘Bola de Oro’ allele (**A**); B: homozygous for TGR-1551 allele (**B**); H: heterozygous; PS: Piel de Sapo control (**C**).

Fifty-seven BC_4_(95)10S_1_ plants and five PS plants were phenotyped for the resistance against race 5 of *Px*. Twelve days after inoculation, the plants of the PS line showed severe infection symptoms. Different levels of fungal sporulation were recorded for the BC_4_(95)10S_1_ plants. Thus, the segregation ratio of the BC_4_(95)10S_1_ plants observed was 34 resistant:23 susceptible, which is not consistent with a 13:3 ratio corresponding to the independent segregation of two genes, one dominant gene, and one recessive gene. These plants were genotyped with the HRM markers *cysdv63*, *cysdv65*, *Oidio3*, *Oidio2*, and *Oidio1*. All the plants were homozygous for the BO allele at the position of the marker *Oidio3*, while segregation was observed for the rest of them. The combined effect of the QTLs in chromosomes 5 and 12 was analyzed (Figure 2). Those plants that were homozygous for the BO allele at the candidate region in chromosome 5 were only resistant when they were homozygous for the TGR-1551 allele at the *locus Oidio2* (Figure 2A). Those plants that were homozygous for the TGR-1551 alleles in the candidate region of chromosome 5 showed mild or no symptoms independently of their genotype at the position of the marker *Oidio2* (Figure 2B). There were 25 heterozygous plants at the position of the markers *cysdv63* and *cysdv65* that were heterozygous or homozygous for the BO allele at the position of the marker *Oidio2* in chromosome 12. Among those plants, there were 10 that showed a susceptible phenotype (Figure 2C). The resistance response of the heterozygous plants might have been affected by the lower temperatures recorded during the assay (the medium temperature was lower than 21 °C).

## 3. Discussion

In this work, advanced backcross lines derived from an initial cross between TGR-1551 and ‘Bola de Oro’ were used to narrow the candidate regions of the resistance genes to *Px* and to develop PCR-based markers tightly linked to this resistance. Moreover, thanks to the genotyping achieved via the sequencing of both parents, candidate resistance genes have been provided.

Resistance to *Px* races 1, 2, and 5 derived from TGR-1551 was initially described as independently controlled by one dominant gene on chromosome 5 and a recessive one [31,32]. The phenotyping of an F_2_ population derived from the same cross and its genotyping through Bulked Segregant Analysis (BSA) and AFLP markers allowed for the identification of two QTLs, namely, *Pm-R1-2* and *Pm-R5*, the first of them related to resistance to races 1 and 2 and the latter to resistance against race 5 [21]. Two resistance-like genes, *MRGH5* and *MRGH63*, belonging to the NBS-LRR (Nucleotide-binding site and leucine-rich repeats) family, were identified as candidate genes for CPM resistance in the TGR-1551 in the region of chromosome 5. Moreover, the molecular markers developed by Yuste-Lisbona et al. [21] were also linked to the resistance derived from WMR29. The use of advanced backcrossed lines and the genotypic information derived from New Generation-Sequencing technologies (NGS) allowed us to narrow the candidate region related to the resistance to the three races to a 251 kb region between the HRM markers *cysdv626* (chr5: 26,058,307 bp) and *cysdv6531* (chr5: 26,309,060 bp). This narrowed candidate region contains 34 predicted genes, of which 31 were annotated (Supplementary Table S3), but does not include *MRGH63* and *MRGH5*, which are approximately <275 kb from the candidate interval (Figure 1). Several QTLs linked to *Px* resistance have been mapped in chromosome 5 (*qPx1-5*, *Pm-AN*, *Pm-w*, *Pm-Edisto47-2*, and *PmV-1-Pi124112*) [14,18,19,20,22,23] but only the QTLs *qPx1-5* and *Pm-AN*, linked to resistance to *Px* race 1 derived from MR-1 and Ano2, respectively, overlapped with the proposed candidate region [19,22] (Figure 1). Moreover, the region in which these resistances are located overlaps with a 760 kb region with the highest concentration of resistance genes in the melon genome [45]. Furthermore, this region has been proven to be highly variable both at the intra- and interspecific levels, explaining the differences in resistance found in different melon genotypes [45].

Most of the annotated genes within the candidate region have resistance-related functions. Among them, there are two genes annotated as “disease resistance proteins” (*MELO3C004320* and *MELO3C031556*), as well as genes related to host pattern recognition receptors (PRR) such as two NBS-LRRs (*MELO3C004319* and *MELO3C031325*) and a “receptor-like cytosolic serine/threonine-protein kinase RBK2” (*MELO3C004315*). The TMV resistance protein N-like, a TIR-NBS-LRR gene possessing homology with resistance genes [46], was frequently annotated (seven of the annotated genes in the candidate interval). As the dominant resistances of powdery mildew identified so far have been easily broken by newly emerging races [47], it has been postulated that many of the QTLs detected could encode PRRs that would be manipulated by the secreted fungal effectors [48,49]. It has recently been proposed that several dominant resistances against powdery mildew in melon could be caused by missense mutations in different LRR-receptor like kinases and TIR-NBS-LRR genes [19,35]. In this sense, two SNPs with a predicted modifier impact were detected within the sequence of a gene coding TMV resistance protein N-like (*MELO3C004311*) (Supplementary Table S4), which could play a potential role in an eventual protein product and thereby confer resistance against *Px*. This TIR-NBS-LRR gene has also been proposed as the candidate gene, derived from MR-1, conferring resistance against *Px* race 1 [19]. Moreover, six variants with modifier impacts were found within the sequence of the predicted gene *MELO3C004297*, which was annotated as a “Branched-chain-amino-acid aminotransferase-like protein” (Supplementary Table S4). These kinds of proteins are related to defense responses in several species. A branched chain amino acid aminotransferase, termed TaBCAT1, has been described as a positive regulator of wheat rust (*Puccinia triticina*) susceptibility, as it had a key role in SA-dependent defense activation [50]. In *A. thaliana*, the levels of the branched-chain amino acid-related compound ILA correlate with the expression of a glucosyltransferase (UGT76B1) that is induced in response to stress [51]. Therefore, these two genes are the principal candidate resistance genes in the proposed interval.

In addition to the previously cited R genes, two predicted genes were noted to have functions related to cell wall formation (*MELO3C002509* and *MELO3C002512*). The cell wall is one of the primary shields against pathogen infections. It acts as a barrier that pathogens must degrade for infection progression, and it also contains antimicrobial compounds [52,53]. Moreover, cell wall compounds that are released during infection can act as damage-associated molecular patterns (DAMPs), triggering plant immune responses thanks to their perception by PRRs [54,55]. It has been proven that a loss of susceptibility factors increases cell wall thickness, leading to a more effective defense response against powdery mildew [28,56,57,58]. Moreover, the resistance derived from TGR-1551 to *Px* could be related to post-inoculation modifications of the cell walls, since, when comparing the response of TGR-1551 and BO against infection, a 50% increase in the number of cells with an accumulation of callose in their cell walls is observed in TGR-1551 [59]. There were also two predicted genes related to photosynthetic processes (*MELO3C004308* and *MELO3C004307*). During a powdery mildew infection, the genes involved in photosynthesis and related processes are upregulated in melons [60]. The candidate region also includes the virus aphid transmission resistance gen (*Vat*) (ten of the predicted genes are annotated as “Vat protein”) [45] carried by TGR-1551 [61]. Moreover, the resistance to CYSDV derived from TGR-1551 has been mapped in the same region [44], which would explain why—while developing a resistant line to CYSDV—the selected plants resulted in being resistant to *Px* and *Aphis gosypii* as well, even though no previous selection against the fungus nor the aphid had been made [62].

Regarding the recessive gene, in a previous work, an RIL population derived from a cross between TGR-1551 and BO was evaluated for resistance to the three races of *Px* and a QTL associated with the recessive gene that was found in chromosome 12 [33]. The SSR markers CMBR111 (chr12: 22,475,952–22,476,042 bp), TJ29 (chr12: 22,475,971–22,476,086 bp), and CMBR150 (chr12: 23,085,299–23,085,068 bp) were found to be linked to this QTL [33]. The same SSR markers were also linked to the resistance QTL(AR5) derived from AR5 [17]. Nevertheless, the phenotyping and genotyping of the advanced backcrossed lines allowed us to narrow the resistance interval to a region of approximately 400 kb between HRM markers *Oidio3* (chr12: 21,870,304 bp) and *Oidio2* (chr12: 22,251,703 bp) (Table 3). This narrowed region is not located within the previously proposed resistance QTL and is also outside the boundaries proposed for other resistance sources such as Edisto27, MR-1, PMR5, WM6, PI 124112, and PI 134198 [17,19,24,25,26,30,35,36,63] (Figure 1). Nevertheless, the recessive gene present in TGR-1551 is unique, since no recessive genes conferring resistance to more than one *Px* race have been reported previously in melons, and this resistance could be conferred by a gene different from those derived from other accessions.

The candidate interval contains 50 predicted genes and all of them have been annotated (Supplementary Table S6). Most of the annotated genes have defense-related functions, which might indicate the presence of a resistance cluster. “Kinase binding” is the molecular function most frequently described, and several predicted genes were also annotated as “cysteine-rich receptor-like protein kinase”. There are two predicted genes annotated as “transcription factors” (*MELO3C002551* and *MELO3C002522*) and another as a “eukaryotic translation initiation factor-like protein” (*MELO3C002515*). Moreover, *MELO3C002553* was annotated as a “sugar carrier protein C-like”. Sugar metabolism plays a key role in the plant–pathogen interaction, as bacteria and fungus tend to acquire these metabolites during infection. Modifications in sugar metabolism during infection plays a key role in the susceptibility to all wheat rust and powdery mildew pathogen species in wheat [64]. In grapevine, Hexose Transporter 5 (VvHT5) is strongly upregulated in coordination with Cell Wall Invertase (VcwINV) during powdery and downy mildew infections [65]. The predicted gene *MELO3C002550* has been annotated as a “Flowering locus T/terminal flower 1-like protein”. Common genetic and epigenetic regulators for flowering and systemic acquired resistance (SAR) have recently been suggested [66]. There is also one predicted gene implicated in photosynthetic processes (*MELO3C002510*).

The effect of the detected SNPs was calculated with SNPeff (Supplementary Table S4). Regarding other accessions, different candidate resistance genes have recently been proposed using a similar approach [19,24,25,35]. There were SNPs affecting the coding region of 19 genes located within the candidate region on chromosome 12. Most of those SNPs were located in sequences of genes coding serine/threonine-protein kinases (STKs) and receptor-like serine/threonine-protein kinases (RLKs) (Supplementary Table S4) and frequently had a predicted modifier impact over the protein function. RLKs are key components of the plant immune system, acting in both broad-spectrum, elicitor-initiated defense responses and as dominant resistance genes in race-specific pathogen defense [67]. Moreover, STK domains not only interact with avirulence proteins but also function as signal transduction mediators [68,69]. Mutations in these proteins could lead to a better recognition of PAMPs or DAMPs and, hence, to a faster plant response against *Px*. A similar phenomenon occurs with serine/threonine-protein kinases, which play a key role in the transduction of the internal and external signals. Some genes coding these kinds of receptors, and located in chromosome 12, have already been proposed as candidate resistance genes derived from MR-1 and PMR5 [19,26,35]. Nevertheless, other candidate genes have been proposed within the same region [24,25]. In addition to the mutations previously described, an SNP was also detected within the coding region of a purine permease. These proteins are involved in cytokinin transport and it has been proven that the suppression of CK transporters can lead to the suppression of the immune response [70]. There was also an SNP that caused the appearance of a new stop codon in the sequence of a gene coding a BTB/POZ domain-containing protein, At3g22104. These proteins are involved in the pathway of protein ubiquitination, and the reduction in ubiquitin levels enhanced the susceptibility to powdery mildew in barley (*Hordeum vulgare)* [71]. Moreover, other components of the ubiquitination complex have also been related to the plant defense against fungi [72,73]. An SNP with a predicted modifier impact was also found within the 3′UTR region of a gene coding a eukaryotic translation initiation factor (EIF). These proteins have been widely described as susceptibility factors in viral infections, but not similar functions have been documented in plant–fungal interactions. Finally, an SNP that caused the loss of a start codon was also detected within the sequence of a gene coding a (3S,6E)-nerolidol synthase 1-like protein. These proteins are involved in monoterpene and sesquiterpene biosynthesis. The enhanced production of some sesquiterpenes has been related to the inhibition of bacterial growth [74], and many monoterpene-induced transcripts are annotated as either transcription factors or defense genes [75].

A comparative transcriptome profiling of genes and pathways related to resistance against powdery mildew was performed in the contrasting melon genotypes MR-1 and Topmark [76]. The differentially expressed genes (DEGs) detected were classified into seven functional groups: pathogen recognition, signal transduction, transcription factors (TFs), phytoalexin biosynthesis, other primary metabolite functions, Mildew Locus O genes (MLOs), and pathogenesis-related (PR) proteins. The defense-related genes showed an increased expression at the early stage of *Px* infection in the resistant genotype MR-1, whereas the defense response was suppressed in the susceptible cultivar. Moreover, the expression changes tended to be maintained during the infection [76]. There are also several non-coding RNAs (long non-coding RNAs (lncRNAs) and circular RNAs (circRNAs)) [77,78] that have been described as being related to *Px* resistance, promoting the expression of genes involved in defense processes. Therefore, the resistance to *Px* in melons comprises many defense-related mechanisms. The regions presented herein as linked to the resistance derived from TGR-1551 contain several genes involved in the detection of fungal elicitors, signal transduction, and resistance response. A transcriptomic analysis would be needed to identify differentially expressed genes in the candidate regions and further VIGs assays will be performed to functionally validate the proposed candidate genes.

Resistance to powdery mildew has been described as temperature-dependent in different species, including melons and cucumbers [59,79,80,81]. The phenotyping/genotyping results of the BC_4_(95)10S_1_ offspring revealed that the resistance conferred by the QTL located in chromosome 5 may decrease under low temperature conditions in heterozygous lines. The plants homozygous for the TGR-1551 allele at *locus Oidio2* were not affected by low temperatures, whereas those plants that were heterozygous for the markers *cysdv63* and *cysdv65* and heterozygous or homozygous for the BO allele at *locus Oidio2* showed a decrease in their resistance response (Figure 2C). This condition had not previously been observed in the assays carried out in the same season of different years with higher temperatures. These results were consistent with those observed by Beraldo-Hoischen et al. [59], where TGR-1551 and the NIL21, carrying the QTL Pm-R derived from TGR-1551, were susceptible when inoculated at low temperatures. Under low temperature conditions, a delay in the resistance response was observed and the number of cells with callose accumulation per point of penetration decreased. A similar behavior was observed in the resistant cucumber line PI 1970088-1, where a major QTL was always detected under high and low temperature conditions and four other QTLs were temperature-dependent [82]. Thus, the resistance conferred by the dominant QTL located in chromosome 5 would be temperature-dependent only for the heterozygous plants, while the temperature would not affect the resistance provided by the recessive QTL located in chromosome 12.

## 4. Materials and Methods

### 4.1. Plant Material

The resistance source used in this study, TGR-1551, is a Zimbabwean melon cultivar described as resistant to *Px* races 1, 2, and 5. Generations used in this study come from the breeding program aimed at the introgression of the resistance derived from TGR-1551 to commercial types via backcrosses to the Spanish melon cultivars ‘Bola de Oro’ and ‘Piel de Sapo’. The populations inoculated were constructed from an original BC_3_ population composed of 200 plants obtained from the F_1_ TGR-1551 x ‘Bola de Oro’ and three successive backcrosses to the susceptible cultivar ‘Bola de Oro’ (BO). BC_3_ plants were selected for carrying the candidate regions previously described as linked to *Px* resistance and with less genetic background from TGR-1551. New backcrosses to BO and self-pollinations were carried out in consecutive years (Supplementary Figure S1). Initially, fifty-two of the BC_3_ plants were genotyped with markers linked to the candidate resistance region to *Px* at chromosome 5 (see details below) in 2018. Twenty BC_3_ plants were selected, backcrossed to BO, and self-pollinated to obtain their corresponding BC_4_ and BC_3_S_1_. In 2019, the BC_3_S_1_ and BC_4_ families generated were genotyped to confirm the presence of the genomic regions associated with resistance to CPM in chromosome 5 and 12. The 20 selected BC_3_S_1_ were artificially inoculated with *P. xanthii*. The corresponding BC_4_ were then backcrossed to BO and to the CPM-susceptible Spanish cultivar ‘Piel de Sapo’ (PS) to obtain BC_5_ and BC_4_ x PS, respectively. In 2020, plants of those populations were genotyped with background and HRM markers to confirm the presence of the genomic regions of interest. Three BC_5_ and four BC_4_ x PS plants were selected and self-pollinated to obtain BC_5_S_1_ and BC_4_ x PS-S_1_ families, respectively. In 2021, the BC_5_S_1_ and BC_4_ x PS-S_1_ families were genotyped with HRM markers and phenotyped for CPM resistance to narrow the genomic locations of the resistance to *Px* (Table 1). BC_5_S_1_-resistant plants that were homozygous for the TGR-1551 allele at the candidate region of chromosome 5 were self-pollinated and their BC_5_S_2_ progenies were phenotyped for CPM resistance.

### 4.2. Artificial Inoculations

The isolates of *Podosphaera xanthii* used in this experiment were SF30 (race 1), P-15.0 (race 2 F), and C8 (race 5). They were all collected in melon crops growing under greenhouses in the South of Spain. For each case, and with the help of a brush, a small amount of conidia was taken from the natural mycelial surface present on the melon leaves and placed on cut and previously disinfected cotyledons of cucumber var. ‘Black Beauty’ growing on water agar medium (0.8% agar, 3% benzimidazole, and 3.2% sucrose) in petri dishes under axenic conditions, as described by Yuste-Lisbona et al. [29]. Cotyledons were disinfected (solution of NaClO at 20 gr/L) before use to allow a prolonged preservation of both cotyledon and mildew, avoiding possible contamination of the powdery mildew colonies. Once the pathogen was identified as *P. xanthii* based on its morphological characters under light microscope examination, monosporic cultures were performed. Natural powdery mildew is composed of a mixture of different genotypes whose competitivity, in particular under in vitro culture conditions, can vary. So, cloning by single conidia isolation seemed to be necessary. An agar-coated eyelash, moistened by medium contact, permitted the straightforward capture of individual conidia under binocular lens, which were then deposited on cotyledons maintained under axenic conditions in individual petri dishes. Colonies appeared after about 15 days under incubation conditions in growth room with controlled temperature (23 °C) and relative humidity (38–65%) at a photoperiod of 16/8 h (light/dark). The races of the different fungal isolates were confirmed by analyzing the reactions of a set of differential melon lines to each isolate, as described by Bardin et al. [8]. Those colonies were then used to carry out the inoculations. The fungus was sub-cultured several times until the inoculation date. The monosporic cultures were maintained under the same conditions of incubation until the inoculation dates.

Three to five plants each from the resistant parental line TGR-1551, the susceptible parental line ‘Bola de Oro’, and their F_1_ progeny, as well as a variable number of plants of the different generations (Supplementary Figure S1), were inoculated with races 1, 2, and 5 of *P. xanthii* on the same leaf. Due to availability of inoculum source and considering that response to the three races in TGR-1551-derived plant materials is homogeneous, two of the BC_4_S_1_ families of interest were inoculated solely with race 5 in 2020.

All plants were inoculated artificially by depositing a small amount of conidia from each race at two spots (at each side of, and equidistant from, the main leaf vein) on the second true leaf of each plant [83]. Inoculation with the three races was carried out on the same leaf of each plant (Supplementary Figure S2).

The plants were maintained and inoculated in an insect-proof glasshouse during spring, from the middle of March to the middle of June, at temperatures ranging from 18 to 32 °C, the relative humidity ranging between 43–79%, and photoperiod ranging from 12:12 to 16:8 h (light:dark). That period of time is the most adequate to evaluate PM because it is the season when melons are grown under greenhouses in Spain as well as along the Mediterranean basin. Twelve days after inoculation, plants were scored according to the level of sporulation of the fungus, using a scale from 0–3, similar to those previously used by other authors for powdery mildew in melons [21,31,84,85,86] (Supplementary Figure S2). The following classes were established: class 0, no conidia germination; class 1, some conidia germination, no visible sporulation, and no disease progression; class 1.5, low level of sporulation, and the disease does not seem to progress; class 2, moderate level of sporulation and sparse mycelia but the disease progresses; class 2.5, clear sporulation; and class 3, profuse sporulation. Plants in classes 0 and 1 were considered resistant (R), those in class 1.5 were considered moderately resistant (MR), and those in classes from 2 to 3, where the infection progressed, were considered susceptible (S).

### 4.3. Molecular Markers and Genotyping Methods

Total DNA was extracted from young leaves following the method described by Doyle and Doyle [87] with minor modifications [39]. DNA was quantified using spectrophotometry via a Nanodrop ND-1000 Spectrophotometer v.3.5 (LabTech International, Heathfield, UK) and adjusted to the concentrations suited for the different genotyping analyses.

Previously existing SNPs and new ones developed in this work were used to genotype the different segregating populations. Initially, the 200 BC_3_ plants were genotyped with an existing panel of 124 SNP markers evenly distributed throughout the genome (Background markers 1) (Supplementary Table S1), implemented in the Agena Bioscience iPLEX^®®^ Gold MassARRAY platform by the Epigenetic and Genotyping unit of the University of Valencia (Unitat Central d’Investigació en Medicina (UCIM), Valencia, Spain). This SNP set had previously been validated in populations derived from ibericus x acidulus melon crosses [39,40,41,42,43]. The selected BC_3_ plants were genotyped with two additional markers sets, CYSDV1 as well as WMV1 and WMV2, also implemented in the Agena Bioscience Platform, which covered the candidate region on chromosome 5 and had been developed in previous studies [43,44] (Supplementary Table S2). To increase the resolution of the candidate region of chromosome 5, the BC_3_S_1_ and BC_4_ progenies of the 20 selected BC_3_ plants were also genotyped with the panel sets CYSDV1 and CYSDV2 [44] (Supplementary Table S2). The lectures of a GBS experiment, conducted to perform genetic diversity studies (including TGR-1551 and BO, among many other genotypes), were mapped to the reference melon genome (v.4.0) using bowtie2 v.2.3.4 [88] and an SNP calling was carried out with Freebayes v.1.3.4 [89]. A new set of 160 markers evenly distributed throughout the genome that allowed us to distinguish between the different melon groups was selected (Supplementary Table S1). These 160 markers provided complementary information to that offered by the previously used set of 124 markers. This new set was also implemented in the Agena Bioscience Platform and used to genotype the BC_4_ x PS, BC_4_S_1_ and BC_5_ progenies. To further narrow the candidate interval in chromosome 12, the marker CMPSNP361 (Supplementary Table S1) and 10 new SNPs, retrieved from the GBS experiment and evenly distributed within the candidate interval, were adapted to a PCR-based protocol for their analysis by high-resolution melting (HRM) (Supplementary Table S5). For a better analysis of the melting curves provided by HRM markers, we avoided selecting SNPs that produce an A/T nucleotide change. These HRM markers were used to genotype selected BC_3_S_1_ and BC_4_ x PS-S_1_ families. The previously designed HRM markers *csydv63* and *cysdv65* [44] were used to genotype the BC_3_S_1_, BC_4_, BC_4_ x PS, BC_4_ x PS-S_1_, BC_5_, and BC_5_S_1_ selected families. To narrow the candidate region in chromosome 5, two additional SNPs were adapted for analysis by HRM (*csydv626* and *cysdv6351*) and they were used to genotype the BC_4_ x PS-S_1_ and BC_5_S_1_ populations. The predicted effect of the SNPs’ variants found between TGR-1551 and BO within the candidate regions was analyzed with SNPeff version 1.3.4 [90].

## 5. Conclusions

Breeding new cultivars resistant to powdery mildew is the most efficient, durable, and environmentally respectful way to fight this pathogen. The African accession TGR-1551 has been reported as being resistant to CPM races 1, 2, and 5. This work has allowed for the narrowing of the candidate intervals of the dominant and recessive QTLs associated with the resistance to CPM derived from TGR-1551 to a region of approximately 250 kb and 381 kb, respectively. The SNP markers provided herein are a useful resource for the introgression of CPM resistance in commercial melon cultivars and several candidate genes have been proposed. Resistance to CPM is strongly affected by the environmental temperature. Thus, the availability of markers tightly linked to the resistance QTLs is essential for accelerating the introgression of CPM resistance into elite cultivars or landraces.

## Figures and Tables

**Table 1:** Genotype for the SNP markers distributed evenly throughout the genome and located in chromosomes 5 and 12 and for the SNPs in the panels CYSDV1, CYSV2, WMV1, and WMV2 for the BC3 plants selected to evaluate their offspring (H: heterozygous; A: homozygous for ‘Bola de Oro’ allele). The genotype for the set CYSDV2 markers has been inferred from the BC3S1 families. The phenotype for the descendants is indicated (SU: susceptible; SE: segregating). Markers for the candidates’ intervals are in bold format highlighted.

				Number of BC_3_ Plant																			
Markers Set	Marker	Chr ^a^	Genomic Position (bp)	15	19	34	64	78	95	96	105	139	141	166	24	28	37	88	89	146	148	159	198
Background1	CMPSNP898	5	256,610	A	A	A	A	A	A	A	H	A	A	H	A	A	A	H	H	n.d	A	A	A
Background1	CMPSNP387	5	1,260,621	A	A	A	H	A	A	A	H	A	A	H	A	A	A	A	H	n.d	A	A	A
Background1	CMPSNP437	5	1,711,810	A	A	A	H	A	A	A	A	A	H	H	A	A	A	A	H	n.d	H	A	A
Background1	CMPSNP726	5	2,373,146	A	H	A	H	A	A	A	A	A	H	H	A	A	A	A	H	n.d	H	A	A
Background1	SSH9G15	5	5,781,400	A	A	A	H	A	A	A	A	A	A	H	H	H	A	A	n.d	n.d	H	A	A
CYSDV1	cysdv10B	5	6,298,039	A	A	A	H	A	A	A	A	A	A	H	H	H	A	A	A	A	H	H	A
CYSDV1	cysdv11	5	9,494,514	A	A	A	H	A	A	A	A	A	A	H	H	H	A	A	A	A	n.d	H	A
Background1	CMPSNP788	5	12,024,140	A	A	A	H	A	A	A	A	A	A	H	H	H	A	A	A	A	n.d	H	A
WMV1	b5wmv2	5	15,026,076	A	A	A	H	A	A	A	A	A	A	H	H	H	A	A	A	A	H	H	A
CYSDV1	cysdv14	5	17,216,441	A	A	A	H	A	A	A	A	A	A	H	H	H	A	A	A	A	n.d	H	A
Background1	60k41.243	5	19,612,771	H	H	A	H	A	H	A	H	A	H	H	A	A	A	A	A	A	n.d	H	A
WMV1	b5wmv3	5	19,968,717	n.d	A	A	H	A	A	A	A	A	n.d	H	H	n.d	A	A	A	A	n.d	H	A
CYSDV1	cysdv17	5	22,544,163	A	A	A	H	A	A	A	A	A	A	H	H	H	A	A	A	A	n.d	H	A
CYSDV1	cysdv18	5	24,125,662	A	H	A	H	A	A	A	A	A	A	H	A	H	A	A	A	A	n.d	H	A
CYSDV1	cysdv19	5	24,192,280	A	H	A	H	A	A	A	A	A	A	H	A	H	A	A	A	A	n.d	H	A
CYSDV1	cysdv21	5	24,427,738	A	H	A	H	A	A	A	A	A	H	H	A	H	A	A	A	H	n.d	H	A
CYSDV1	cysdv22	5	24,434,940	A	H	A	H	A	A	A	A	A	H	H	A	H	A	A	A	H	n.d	H	A
CYSDV2	cysdv40	5	24,474,795	A	H	A	H	A	A	A	A	A	H	H	A	H	A	A	A	H	n.d	H	A
CYSDV2	cysdv42	5	24,608,464	A	H	A	H	A	A	A	A	A	H	H	A	A	A	A	A	H	n.d	H	A
CYSDV2	cysdv43	5	24,678,113	A	H	A	H	A	A	A	A	A	H	H	A	A	A	A	A	H	n.d	H	A
CYSDV2	cysdv44	5	24,704,705	A	H	A	H	A	A	A	A	A	H	H	A	A	A	A	A	H	n.d	H	A
CYSDV2	cysdv45	5	24,761,527	A	H	A	H	A	A	A	A	A	H	H	A	A	A	A	A	H	n.d	H	A
CYSDV2	cysdv46	5	24,778,081	A	H	A	H	A	A	A	A	A	H	H	A	A	A	A	A	H	n.d	H	A
CYSDV2	cysdv48	5	24,842,569	A	H	A	H	A	A	A	A	A	H	H	A	A	A	A	A	H	n.d	H	A
CYSDV2	cysdv49	5	24,842,825	A	H	A	H	A	A	A	A	A	H	H	A	A	A	A	A	H	n.d	H	A
WMV2	SNP25	5	24,898,321	A	H	A	H	A	A	A	A	A	H	H	A	A	A	A	A	H	A	H	A
WMV2	SNP26	5	25,045,765	H	H	A	H	A	A	A	A	A	H	H	A	A	A	A	A	H	n.d	H	A
CYSDV2	cysdv50	5	25,052,002	H	H	A	H	A	A	A	A	A	H	H	A	A	A	A	A	H	n.d	H	A
CYSDV2	cysdv51	5	25,132,216	H	H	A	H	A	H	A	A	A	H	H	A	A	A	A	A	H	n.d	H	A
WMV1	b5wmv4	5	25,132,292	H	H	A	H	A	H	A	A	A	H	H	A	A	A	A	A	H	n.d	H	A
WMV1	b5wmv4A	5	25,144,133	H	H	A	H	A	H	A	A	A	H	H	A	A	A	A	A	H	n.d	H	A
CYSDV2	cysdv53	5	25,144,085	H	H	A	H	A	H	A	A	A	H	H	A	A	A	A	A	H	n.d	H	A
CYSDV2	cysdv54	5	25,210,212	H	H	A	H	A	H	A	A	A	H	H	A	A	A	A	A	H	n.d	H	A
CYSDV2	cysdv55	5	25,210,573	H	H	A	H	A	H	A	A	A	H	H	A	A	A	A	A	H	n.d	H	A
CYSDV2	cysdv56	5	25,233,221	H	H	A	H	A	H	A	A	A	H	H	A	A	A	A	A	H	n.d	H	A
CYSDV2	cysdv57	5	25,341,873	H	H	A	H	A	H	A	A	A	H	H	A	A	A	A	A	H	n.d	H	A
CYSDV2	cysdv58	5	25,356,079	H	H	A	H	A	H	A	A	A	H	H	A	A	A	A	A	H	n.d	H	A
CYSDV2	cysdv59	5	25,435,244	H	H	A	H	A	H	A	A	A	H	H	A	A	A	A	A	H	n.d	H	A
WMV1	b5wmv5	5	25,694,699	H	H	A	H	A	H	A	A	A	H	H	A	A	A	A	A	H	n.d	H	A
CYSDV2	cysdv60	5	25,744,140	H	H	A	H	A	H	A	A	A	H	H	A	A	A	A	A	H	n.d	H	A
CYSDV2	cysdv61	5	25,756,801	H	H	A	H	A	H	A	A	A	H	H	A	A	A	A	A	H	n.d	H	A
CYSDV2	cysdv61	5	25,772,357	H	H	A	H	A	H	A	A	A	H	H	A	A	A	A	A	H	n.d	H	A
CYSDV2	cysdv62	5	25,776,015	H	H	A	H	A	H	A	A	A	H	H	A	A	A	A	A	H	n.d	H	A
**CYSDV2**	**cysdv63**	**5**	**25,782,654**	**H**	**H**	**A**	**H**	**A**	**H**	**A**	**A**	**A**	**H**	**H**	**A**	**A**	**A**	**A**	**A**	**H**	n.d	**H**	**A**
**Background1**	**CMPSNP464**	**5**	**26,405,006**	**H**	**H**	**A**	**H**	**A**	**H**	**A**	**H**	**A**	**A**	**H**	**A**	**A**	**A**	**A**	**A**	**n.d**	**A**	**H**	**H**
**CYSDV2**	**cysdv65**	**5**	**26,408,895**	**H**	**H**	**A**	**H**	**A**	**H**	**A**	**H**	**A**	**A**	**A**	**A**	**A**	**A**	**A**	**A**	**A**	n.d	**H**	**H**
CYSDV2	cysdv69	5	26,467,357	H	H	A	H	A	H	A	H	A	A	A	A	A	A	A	A	A	n.d	H	H
CYSDV1	cysdv24	5	26,547,204	H	H	A	A	A	H	A	H	A	A	A	A	A	A	A	A	A	n.d	H	H
WMV1	b5wmv7	5	26,592,732	H	H	A	A	A	H	A	H	A	A	A	A	A	A	A	A	A	n.d	H	H
WMV1	b5wmv7B	5	26,592,732	H	H	A	A	A	H	A	H	A	A	A	A	A	A	A	A	A	n.d	H	H
CYSDV1	cysdv23	5	26,752,698	H	H	A	A	A	H	A	H	A	A	A	A	A	A	A	A	A	n.d	H	H
CYSDV1	cysdv25	5	26,769,546	H	H	A	A	A	H	A	H	A	A	A	A	A	A	A	A	A	n.d	H	H
CYSDV1	cysdv26	5	26,940,168	H	H	A	A	A	H	A	H	A	A	A	A	A	A	A	A	A	n.d	H	H
CYSDV1	cysdv27	5	26,957,159	H	H	A	A	A	H	A	H	A	A	A	A	A	A	A	A	A	n.d	H	H
WMV1	b5wmv8	5	26,963,176	H	H	A	A	A	H	A	H	A	A	A	A	A	A	A	A	A	n.d	H	H
CYSDV1	cysdv28	5	27,118,062	H	H	A	A	A	H	A	H	A	A	A	A	A	A	A	A	A	n.d	H	H
WMV1	b2wmv9	5	27,273,256	H	H	A	A	A	H	A	H	A	A	A	A	A	A	A	A	A	n.d	H	H
WMV1	b2wmv10	5	27,464,146	H	H	A	A	A	H	A	H	A	A	A	A	A	A	A	A	A	n.d	H	H
WMV2	SNP29	5	27,538,308	H	H	A	A	A	H	A	H	A	A	A	A	A	A	A	A	A	n.d	H	H
WMV1	b5wmv11	5	27,570,154	H	H	A	A	A	H	A	H	A	A	A	A	A	A	A	A	A	n.d	H	H
CYSDV1	cysdv30B	5	27,570,488	H	H	A	A	A	H	A	H	A	A	A	A	A	A	A	A	A	n.d	H	H
Background1	AI_13-H12	5	28,039,739	H	H	A	A	H	A	A	H	A	A	A	A	A	A	A	A	A	A	H	H
Background1	CMPSNP385	12	344,819	A	A	A	A	A	H	A	A	A	A	A	A	A	A	A	H	n.d	A	A	A
Background1	CMPSNP310	12	5,032,799	A	A	A	A	A	H	A	A	A	A	A	A	A	A	A	A	n.d	A	A	H
Background1	AI_35-A08	12	12,750,025	A	A	A	A	H	A	A	A	A	A	A	A	A	A	A	A	n.d	A	A	H
Background1	ai09g07	12	16,532,245	A	A	A	A	H	A	A	A	A	A	A	A	A	A	A	A	n.d	A	A	H
**Background1**	**CMPSNP285**	**12**	**20,421,309**	**A**	**A**	**H**	**A**	**H**	**H**	**A**	**A**	**H**	**H**	**A**	**A**	**A**	**A**	**H**	**A**	**n.d**	**A**	**A**	**H**
**Background1/2**	**CMPSNP361**	**12**	**23,000,406**	**A**	**A**	**H**	**A**	**A**	**H**	**H**	**H**	**H**	**H**	**A**	**A**	**A**	**A**	**H**	**H**	**A**	**H**	**A**	**A**
Background1	CMPSNP5	12	24,246,762	A	A	H	A	A	H	H	A	A	H	A	H	A	A	A	H	n.d	H	A	A
Background1	fr14f22	12	25,050,570	H	A	A	A	A	H	H	A	A	H	A	H	A	A	A	H	n.d	A	A	A
Background1	P02.03	12	25,661,792	A	A	A	A	A	A	A	A	A	A	A	A	A	A	A	A	n.d	A	A	A
				SE	SE	SE	SE	SE	SE	SE	SE	SE	SE	SE	SU	SU	SU	SU	SU	SU	SU	SE	SU

^a^: chromosome.

**Table 2:** Genotype for SNP markers in the candidate region of chromosome 5 of those BC_4_xPS-S1 and BC5S1 plants that were recombinant between markers cysdv63 and cysdv65 (A: homozygous for ‘Bola de Oro’ allele; H: heterozygous). The phenotype of the genotyped plants is indicated (SU: susceptible). The physical position of the SNPs in version v.4.0 of the melon genome (available at https://melonomics.net/ (accessed on 1 June 2022)) is indicated.

Marker Name	Chr ^a^	Genomic Position (bp) v.4.0	BC_4_(159)4xPS-7S1-13	BC_4_(159)4xPS-7S1-2	BC_5_(159)4-14S1-5
Cysdv63	5	25,782,654	H	H	H
Cysdv626	5	26,058,307	H	H	H
Cysdv6531	5	26,309,060	A	H	A
Cysdv65	5	26,408,895	A	A	A
Phenotype			Resistant	Resistant	Susceptible

^a^: chromosome

**Table 3:** SNP markers used to narrow the candidate interval of chromosome 12 (A: homozygous for the ‘Bola de Oro’ allele; H: heterozygous). The molecular markers in the candidate regions are in bold format. The position of the SNPs according to the last version of the genome (v.4.0, available at http//www.melonomics.net accessed on 1 June 2022) is indicated. The phenotype of the genotyped plants is indicated (SU: susceptible).

			BC_4_(95)10xPS Plants	
Marker	Chr ^a^	Genomic Position v.4.0 (bp)	3	5
*S5_23380459*	5	24,192,281	A	A
***cysdv63***	5	**25,782,654**	**A**	**A**
***cysdv65***	5	**26,408,896**	**A**	**A**
*S5_25653869*	5	26,419,648	A	A
*S12_18721450*	12	18,530,394	A	A
***Oidio3***	**12**	**21,870,304**	**A**	**A**
***S12_22130778***	**12**	**21,923,406**	**A**	**A**
***Oidio 2***	**12**	**22,251,703**	**H**	**H**
*Oidio1*	12	22,309,972	H	H
*CMPSNP361*	12	23,000,406	H	H
Phenotype			SU	SU

^a^: chromosome.

**Table 4:** Genotype for SNP markers in the candidate region of chromosome 12 for the BC3 plants selected for not having a TGR-1551 introgression at chromosome 5 (A: homozygous for ‘Bola de Oro’ allele; H: heterozygous). The phenotype of the BC3S_1_ progenies is indicated (SU: susceptible; SE: segregating). Markers in the candidate interval are in bold format.

			Number of BC_3_ Plant					
Marker	Chr ^a^	Genomic Position (bp)	34	96	139	88	89	148
*CMPSNP285*	12	20,421,309	H	A	H	H	A	A
*Oidio8*	12	20,920,054	H	A	A	n.d ^1^	A	A
*Oidio5*	12	21,470,961	H	H	A	n.d	A	A
*Oidio4*	12	21,866,445	H	H	A	n.d	A	A
***Oidio3***	**12**	**21,870,304**	**H**	**H**	**A**	**A**	**A**	**A**
***Oidio2***	**12**	**22,251,703**	**H**	**H**	**H**	**A**	**A**	**A**
***Oidio1***	**12**	**22,309,972**	**H**	**H**	**H**	**A**	**A**	**A**
***OidioC***	**12**	**22,875,711**	**H**	**H**	**H**	**H**	**A**	**H**
*OidoB*	12	22,913,643	H	H	H	H	H	H
*OidioA*	12	22,915,510	H	H	H	H	H	H
*CMPSNP361*	12	23,000,406	H	H	H	H	H	H
**Phenotype**			SE	SE	SE	SU	SU	SU

^a^: chromosome. ^1^: missing data.

## Supplementary Materials

The supporting information can be downloaded at: https://www.mdpi.com/article/10.3390/ijms232012553/s1.
