# Supplementary material for: Advanced Genetic Studies on Powdery Mildew Resistance in TGR-1551
Source: Int J Mol Sci. 2022 Oct 19;23(20):12553. doi: 10.3390/ijms232012553 (PMC9604395; doi:10.3390/ijms232012553)
Supplement: Supplementary file 1 [file ijms-23-12553-s001.zip › Suplementario_Figure_S1_Plant material generated and methodology used_comentariosMAria.pptx]

## Slide 1
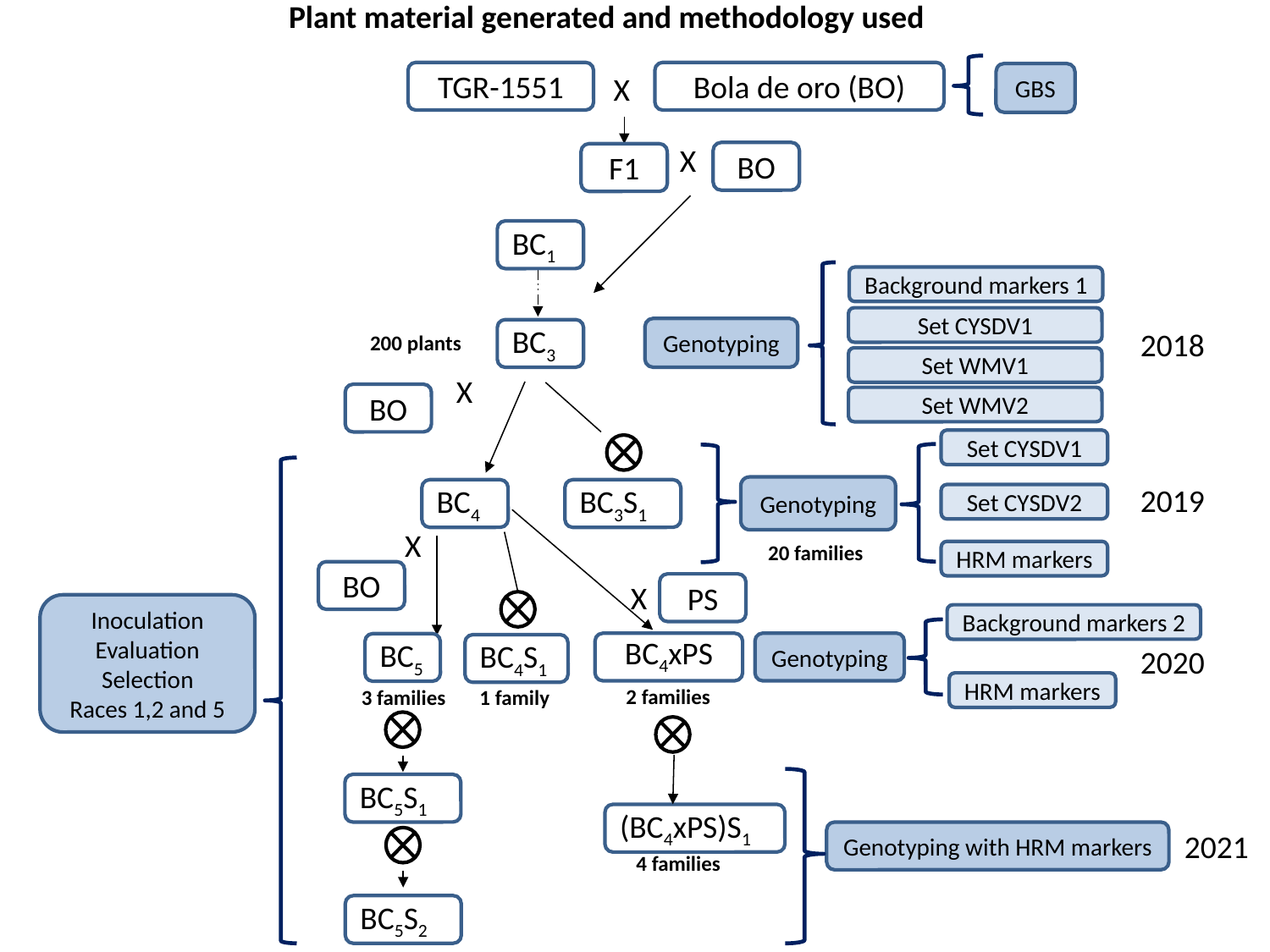

# Plant material generated and methodology used
TGR-1551
Bola de oro (BO)
X
X
BO
F1
BC1
BC3
200 plants
GBS
Background markers 1
Set CYSDV1
2018
Genotyping
Set WMV1
X
BO
Set WMV2
Set CYSDV1
2019
Genotyping
BC4
BC3S1
Set CYSDV2
X
20 families
HRM markers
BO
X
PS
Inoculation
Evaluation
Selection
Races 1,2 and 5
Background markers 2
Genotyping
BC4xPS
BC5
BC4S1
2020
HRM markers
2 families
3 families
1 family
BC5S1
(BC4xPS)S1
2021
Genotyping with HRM markers
4 families
BC5S2
