# Supplementary figures and images for: Advanced Genetic Studies on Powdery Mildew Resistance in TGR-1551
Source: Int J Mol Sci. 2022 Oct 19;23(20):12553. doi: 10.3390/ijms232012553 (PMC9604395; doi:10.3390/ijms232012553)

## Slide 1
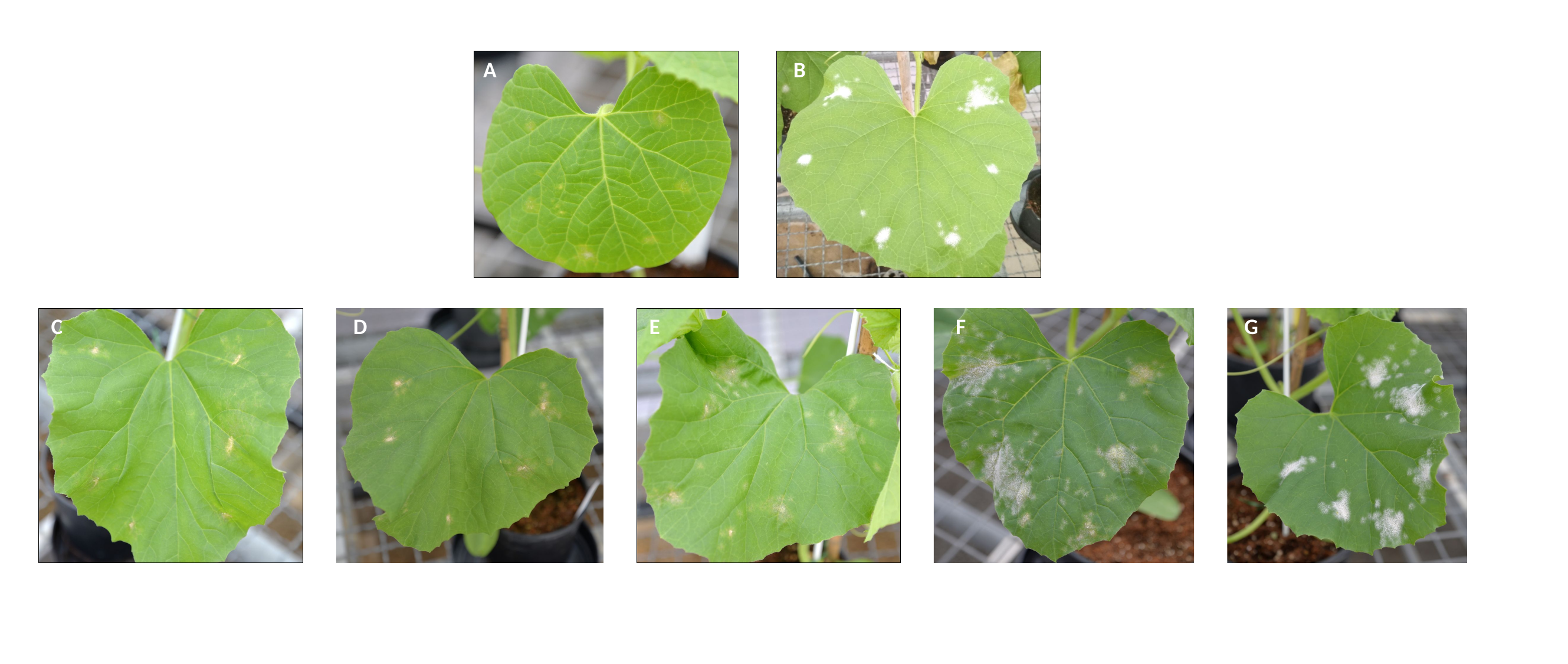

A
B
B
C
D
E
F
G

Supplement: Supplementary file 1 [file ijms-23-12553-s001.zip › Suplementario_Figure_S2_SINTOMAS_OIDIO.pptx]
